# High-Throughput Sequencing Facilitates Discovery of New Plant Viruses in Poland

**DOI:** 10.3390/plants9070820

**Published:** 2020-06-29

**Authors:** Julia Minicka, Aleksandra Zarzyńska-Nowak, Daria Budzyńska, Natasza Borodynko-Filas, Beata Hasiów-Jaroszewska

**Affiliations:** 1 Department of Virology and Bacteriology, Institute of Plant Protection–National Research Institute, Wegorka 20, 60-318 Poznan, Poland; A.Zarzynska@iorpib.poznan.pl (A.Z.-N.); D.Budzynska@iorpib.poznan.pl (D.B.); 2 Plant Disease Clinic and Bank of Pathogens, Institute of Plant Protection–National Research Institute, Wegorka 20, 60-318 Poznan, Poland; N.Borodynko@iorpib.poznan.pl

**Keywords:** virus, virus identification, high-throughput sequencing, phylogenetic analysis

## Abstract

Viruses cause epidemics on all major crops of agronomic importance, and a timely and accurate identification is essential for control. High throughput sequencing (HTS) is a technology that allows the identification of all viruses without prior knowledge on the targeted pathogens. In this paper, we used HTS technique for the detection and identification of different viral species occurring in single and mixed infections in plants in Poland. We analysed various host plants representing different families. Within the 20 tested samples, we identified a total of 13 different virus species, including those whose presence has not been reported in Poland before: clover yellow mosaic virus (ClYMV) and melandrium yellow fleck virus (MYFV). Due to this new finding, the obtained sequences were compared with others retrieved from GenBank. In addition, cucurbit aphid-borne yellows virus (CABYV) was also detected, and due to the recent occurrence of this virus in Poland, a phylogenetic analysis of these new isolates was performed. The analysis revealed that CABYV population is highly diverse and the Polish isolates of CABYV belong to two different phylogenetic groups. Our results showed that HTS-based technology is a valuable diagnostic tool for the identification of different virus species originating from variable hosts, and can provide rapid information about the spectrum of plant viruses previously not detected in a region.

## 1. Introduction

Viruses cause significant yield and quality losses in a wide variety of cultivated crops. Increasing international travel and the trade of plant material, as well as climate changes, enhance the risk of spreading and introducing new viruses and their vectors into production systems [1,2]. Moreover, most of the viruses are multihost pathogens with a wider range of hosts, rather than the specialists (and, therefore, the host range of many viruses overlap). Hence, a single plant might be infected by different unrelated viral species [3,4,5]. The interactions between viruses coinfecting the same host might affect their host range, transmission rate, virus accumulation and, as a consequence, the presence and intensity of symptoms [6,7,8,9]. 

Moreover, plant viruses can emerge in crops from reservoir wild plant hosts in which they are often asymptomatic. Spread from the reservoirs into a new environment with the establishment of productive infections and effective between-host transmission mechanism are steps that require emergence to occur [10]. In addition, many wild plants appear to have multiple infections, including both acute and persistent viruses [11]. At present, novel viruses are being discovered rapidly in wild hosts in diverse natural ecosystems, and this discovery has been accelerated by metagenomics techniques that permit the sequencing of putative viral nucleic acids without a prior knowledge about the present viruses or associated host organisms [12].

Most of the viruses infecting plants are RNA viruses, whose mutation rate is very high [13]. Appropriate diagnosis is especially important in positive-sense single-stranded RNA ((+)ssRNA) viruses, which are characterised by a small genome, fast replication rate, and a lack of repair mechanisms and, therefore, a great potential for genetic differentiation [14]. This allows the maintenance of the genetic diversity of the viral populations and the adaptation to the ever-changing environment while simultaneously disrupting precise targeted diagnostics approaches.

The use of the appropriate diagnostic method is crucial in maintaining healthy material, preventing the spread of the diseases, and carrying out phytosanitary measures. A traditional diagnostic method using transmission electron microscopy (TEM) allows only for the morphological observation of particles in leaf sap derived from infected plants [15]. The standard diagnostic tests (ELISA assay, PCR, RT-PCR, qRT-PCR), despite their potential sensitivity and specificity, require specific primers or sera, and thus specific knowledge about the diagnosed pathogen and its group or family [16,17,18,19]. A huge problem in the precise diagnosis of viruses is the appearance of the new genetic variants by mutation, reassortment or recombination that can significantly differ from the parental viral particle [20]. Furthermore, the diagnosis of mixed infections is associated with many problems, mostly due to the presence of individual viral components in a higher or lower concentration [21]. 

High-throughput sequencing (HTS) is a rapidly developing technique, providing novel opportunities for diagnosis and epidemiology. This technique allows for the sequencing of millions of nucleotides in a short time, which enables the detection of the most viral pathogens in the sample [22]. Moreover, HTS does not require any prior information about pathogens before sequencing [23,24,25]. Due to the possibility of sequencing millions of nucleotide sequences, it can deliver a global spectrum of occurring strains or species of pathogens. In combination with the bioinformatic analysis of the obtained raw data, it makes possible to detect all known pathogens and discover new ones from symptomatic or asymptomatic plants, as well as substrates, e.g. water or soil [26]. HTS was first used in 2009 for the detection of plant viruses, and since then, it has often been used as a diagnostic tool [25,27,28,29,30]. Therefore, it is a good alternative to other diagnostic tests, especially in the context of unexpected or unknown viruses that might be potential threats to plant health. Knowledge of the occurrence and degree of infestation of crops, weeds, trees and ornament plants allows the introduction of appropriate regulations, and thus prevents the occurrence of an epidemic, as well as allowing for the development of new strategies for plant protection. 

In this study, an HTS-based approach was applied for the detection and identification of different viruses from infected plant material collected in Poland. To this end, various plants from different families were used, including cultivated and ornamental plants, weeds and trees. By following this procedure, we successfully detected new pathogens for Poland, identified mixed infections, and obtained the exact genetic characteristic of the pathogens. With the advantage of HTS methods that have been developed to look for virus-like sequences without the bias of only looking for known viruses, we were able to identify virus species that have not been detected using conventional RT-PCR. Furthermore, the phylogenetic analysis was performed in order to establish the genetic relationships of: melandrium yellow fleck virus, clover yellow mosaic virus and cucurbit aphid-borne yellows virus, identified in this study with others described to date.

## 2. Results

### 2.1. Bioassay and Electron Microscopy

**Figure 1:**
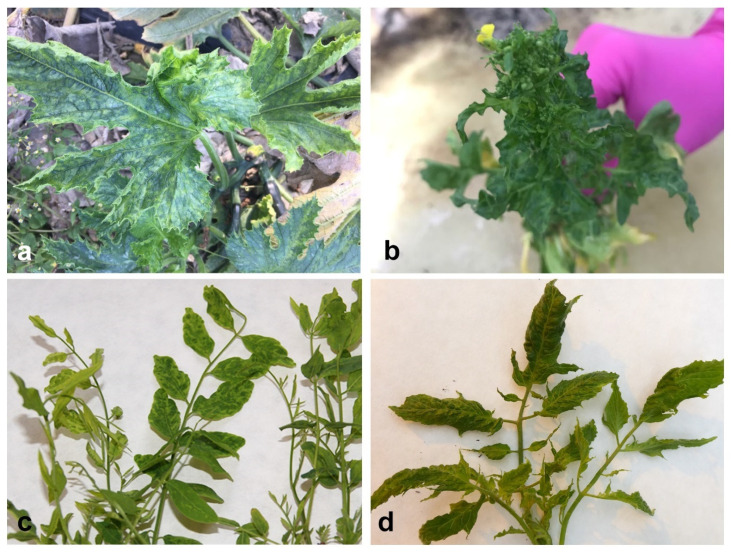
Different disease symptoms on collected plants: (**a**) deformation and chlorotic changes of leaf blades of *C. pepo* convar. giromontiina (Sample 40—Table S1); (**b**) reduction of growth and leaf stunting of *R.* × *prostrata* (Sample 11—Table S1); (**c**) chlorotic mosaic on *R. pseudoacacia* (Sample 4—Table S1); (**d**) deformation of leaves of *S. lycopersicum* (Sample 22—Table S1).

During the surveys performed in Poland in 2018–2019, 50 samples from plants belonging to different botanical families were collected. In all cases, various disease symptoms, in the form of leaf blade deformations of varying severity, discoloration, chlorosis, necrosis of leaf blade, and growth reduction, were observed (Table S1). Cucurbita pepo convar. giromontiina Greb. and Cucumis sativus L. plants were characterised by strong deformation and chlorotic mosaic of leaf blades, often accompanied by fruit necrosis (Figure 1a). The symptoms on weeds (Verbena officinalis L., Silene latifolia Poir. and Rorippa × prostrata (J. P. Bergeret) Schinz et Thell.) were in a form of leaf deformation and the stunting of plants (Figure 1b), whereas on Robinia pseudoaccacia L. the deformation of leaves, the presence of chlorotic mosaics and leaf stunting were observed (Figure 1c). The characteristic features of Solanum lycopersicum L. plants were deformations of leaf blades (Figure 1d), leaf discoloration, and the presence of necrotic spots on leaf blades. On Vicia faba L. cv. minor, chlorotic mosaic on leaf blades was visible, sometimes accompanied by necrotic changes. In the case of ornamental plants, necrotic lesions on leaves and stem were observed on Chrysanthemum multiflorum Ramat., necrotic lesions on stem on Gerbera jamesonii Bolus. plants, and leaf deformation on Delphinum beladonna L. 

**Figure 2:**
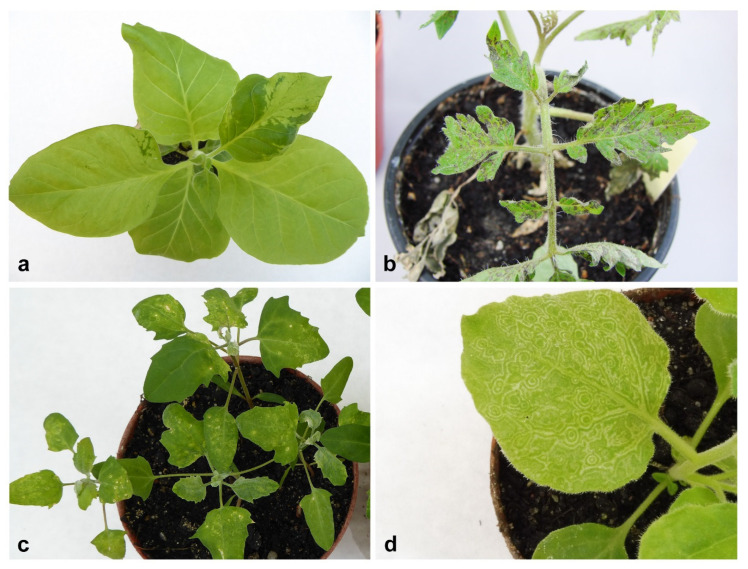
Different disease symptoms on test plants after 10 dpi: (**a**) discoloration and deformation of leaf blades of *N. tabacum* cv. Xanthi (sample 37—Table S1); (**b**) necrotic changes on *S. lycopersicum* cv. Betalux (sample 23—Table S1); (**c**) chlorotic spots on *C. quinoa* (sample 41—Table S1); (**d**) chlorotic mosaic on *N. benthamiana* (sample 12—Table S1).

All samples were transferred to the test plants, and 10 days post-inoculation (dpi) positive result of bioassay was observed for 45 original plants. The symptoms observed on particular host species and test plants were summarised in the Supplementary file (Table S1). Symptoms included deformations of leaf blades, mosaics, chlorotic or necrotic spots on leaf blades, or weakness of the plants and reduction of growth (Figure 2). Zucchini and cucumber samples after transmission to test plants displayed symptoms on *Nicotiana tabacum* L. cv. Xanthi, *C. pepo* convar. giromontiina and *Chenopodium quinoa* Willd. In the case of tobacco plants (sample 37—Table S1), the symptoms were very strong in the form of deformations and discoloration of leaves (Figure 2a). Zucchini samples were also characterised by the presence of chlorotic spots (Figure 2c), local chlorotic spots on leaf blades of *C. quinoa* and severe chlorotic mosaic on *C. pepo* convar. giromontiina. Samples derived from *S. lycopersicum* caused the disease symptoms on all test plants. In two samples (sample 23 and sample 25—Table S1), after transferring to test tomato plants, the necrosis changes were visible 10 days after inoculation (Figure 2b), leading to the death of plants in the next 10 days of observation. On *N. benthamiana* L. and *N. tabacum* cv. Xanthi after inoculation from tomato (sample 23 and sample 25), the symptoms were in the form of leaf blade deformations and chlorosis. *V. faba* cv. minor after performing the bioassay gave disease symptoms only on *C. quinoa*, in the form of chlorotic spots (sample 13). Samples derived from *R. pseudoacacia* were characterised by the presence of local necrotic spots and necrotic ringspots on *C. quinoa*, and leaf deformation and necrotic ringspots on *N. tabacum* cv. Xanthi (sample 2, 4, 7, 8—Table S1). *G. jamesonii* and *C. multiflorum*, belonging to ornamental plants, showed a similar pattern of disease symptoms in the bioassay, mostly in a form of local necrotic spots on *S. lycopersicum*, *N. tabacum* cv. Xanthi and *C. quinoa*, and leaf deformation and chlorotic spots on *N. benthamiana*. In the sample 12, derived from *D. belladonna* (ornamental plant) after inoculation to test plants, the symptoms in a form of chlorotic mosaic on *N. benthamiana* (Figure 2d), and leaf chlorosis on *C. quinoa* were observed. No symptoms were observed after the inoculation of tomato and tobacco plants with the sap from collected weeds. Symptoms mainly occurred on *N. benthamiana* in the form of leaf deformities (sample 10 and 11—Table S1) and in a form of chlorosis on *C. quinoa* (sample 9—Table S1).

**Figure 3:**
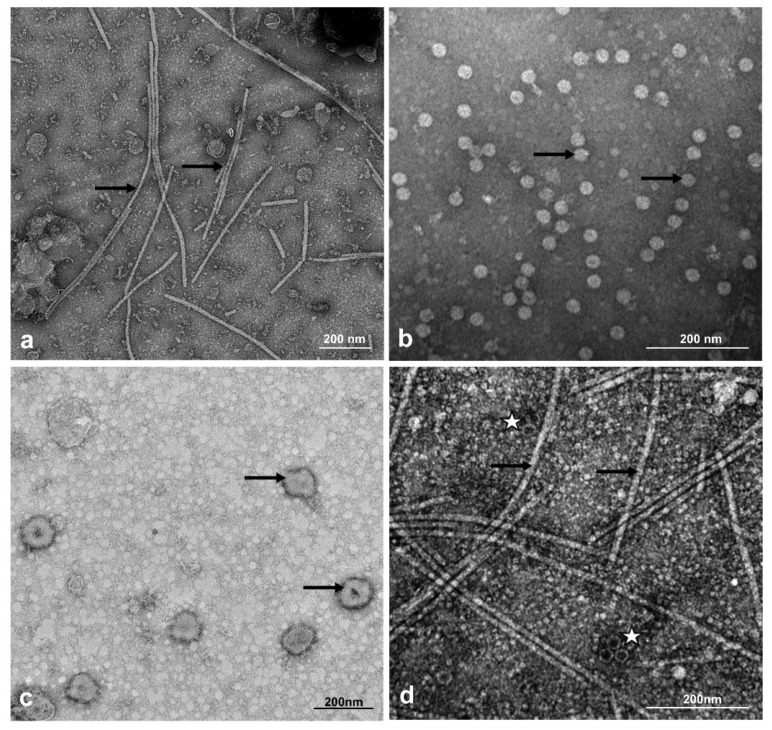
Different types of viral particles observed in leaf sap from infected plants: (**a**) filamentous PepMV particles of about 530 nm in length (arrows) in leaf sap from *S. lycopersicum* (Sample 18); (**b**) isometric CMV particles of about 30 nm in diameter (arrows) in leaf sap from *C. pepo* convar. giromontiina (Sample 48); (**c**) isometric TYRV particles of about 80–100 nm in diameter (arrows) derived from *C. multiflorum* (Sample 29), (**d**) mixed infection of filamentous PepMV particles of about 530 nm (arrows) and isometric CMV particles about 30 nm in diameter (asterisks) from *S. lycopersicum* plants (Sample 20). Bar = 200 nm.

After the examination of the collected samples under the transmission electron microscope (TEM), the presence of various viral particles, both isometric and filamentous, of different lengths and diameters was observed. In tomato samples, mainly filamentous virus particles about 530 in length, characteristic for the *Potexvirus* genus, were present (Figure 3a). Additionally, in three tomato samples, the presence of these filamentous particles was accompanied by the presence of filamentous virus particles about 700 nm in length, isometric particles about 30 nm in diameter (Figure 3d), or isometric particles about 80–100 nm in diameter, indicating the presence of mixed infections. In the samples derived from zucchini and cucumbers, isometric particles of about 25–30 nm in diameter (Figure 3b), or filamentous particles with a length of about 750 nm, were observed. These particles occurred individually or in a complex. Mixed infection of these particles was observed in three zucchini and two cucumber plants. Samples from trees and shrubs (*R. pseudoacacia*, *Sambucus nigra* L.) were characterised by the presence of isometric particles of about 30 nm in diameter, whereas in samples derived from ornamental plants (*G. jamesonii* and *C. multiflorum*), the presence of isometric particles with a diameter of about 80–100 nm was observed (Figure 3c).

### 2.2. RT-PCR Detection

RT-PCR reactions were performed for all the collected samples and the positive results were obtained for 29 of them (Table S1). In three samples from *R. pseudoacacia*, one from *S. nigra* and one from *V. faba* cv. minor, no symptoms were observed on the test plants, no particles were observed at TEM, and the RT-PCR assay was also negative. These samples were not subjected to further analysis. In tomato samples, mostly pepino mosaic virus (PepMV, *Potexvirus* genus, Alphaflexiviridae family) was detected, but the presence of cucumber mosaic virus (CMV, Cucumovirus genus, Bromoviridae family) or potato virus Y (PVY, Potyvirus genus, Potyviridae family) has also been noticed (Table S1). On plants from the Cucurbitaceae family, infections with CMV, watermelon mosaic virus (WMV, Potyvirus genus, Potyviridae family) and zucchini yellow mosaic virus (ZYMV, Potyvirus genus, Potyviridae family) were detected, with a predominance of WMV in 7 out of 17 analysed samples. In *V. faba* cv. minor no viruses were detected by RT-PCR tests. In four samples from *R. pseudoacacia* and two from *S. nigra*, which represent trees and shrubs, we observed positive results with universal primers for nepo B viruses. Therefore, we performed additional RT-PCR for the presence of tomato black ring virus (TBRV, Nepovirus genus, Secoviridae family), which confirmed the presence of this virus in the tested samples (Table S1). In ornamental plants (*G. jamesonii* and *C. multiflorum*), the presence of TSWV was observed, with mixed infection with CVB in a sample derived from chrysanthemum (Table S1). In *D. belladonna* (ornamental plant), we did not identify any of the tested viruses. In 16 original samples, six of *C. pepo* convar. giromontiina, two of *C. sativus*, two of *S. lycopersicum*, one of *R. pseudoacacia*, one of *V. officinalis*, one of *S. latifolia*, one of *R.* × *prostrata*, one of *D. belladonna* and one of *V. faba* cv. minor, despite the symptoms on test plants and the presence of virus particles in TEM, we did not identify any viruses using selected RT-PCR tests. These samples, together with three zucchini samples (sample 41, 44, 45—Table S1) and 1 sample from tomato (sample 21—Table S1) that were RT-PCR positive, but gave an unusual pattern of symptoms on infected plants, were further analysed by HTS.

### 2.3. HTS for Identification of the Viruses in Selected Samples

Multiple virus species in single or mixed viral infections were found in the 20 samples analysed by HTS. At least one viral species was detected in each of the analysed samples. Mixed infections were identified in 10 of 20 analysed samples. A total number of reads and the number of reads mapped for the individual virus reference sequences are summarised in Table 1. Raw sequence reads were deposited in the NCBI Sequence Read Archive (SRA) under BioProject accession number PRJNA590139. The analysis enabled the identification of the assembled contigs of 13 different viruses, some of which covered a large extension of the viral genomes, comprising their near complete or complete genomes. 

In two weed samples (sample 2 and 8—Table 1), we identified the viruses that had not previously been found in Poland: clover yellow mosaic virus (ClYMV) (*Potexvirus* genus, Alphaflexiviridae family) on *V. officinalis* plants, and melandrium yellow fleck virus (MYFV) (Bromovirus genus, Bromoviridae family) on *S. latifolia* plants. The HTS analysis of the third weed (*R.* × *prostrata*–sample 16) revealed the presence of turnip mosaic virus (TuMV, Potyvirus genus, Potyviridae family) in the sample (Table 1). 

In samples derived from Cucurbitaceae crops, the bioinformatic analysis of HTS data revealed the presence of mixed infections of commonly occurring viruses, such as ZYMV, WMV and CMV, as well as cucurbit aphid-borne yellows virus (CABYV, Polerovirus genus, Luteoviridae family) and cucumber leaf spot virus (CLSV, Aureusvirus genus, family Tombusviridae). Reads mapped to the CLSV reference sequence (NC_007216) were detected in one sample (Table 1) and accounted for 0.0015 % of the total number of reads for this sample, which covered the near complete (99.2%) genomic sequence of the virus.

The data analysis of high-throughput Illumina sequences from tomato plants revealed the presence of the PepMV, in complex with tomato yellow ring virus (TYRV, Orthotospovirus genus, Tospoviridae family), or CMV (Table 1).

In *V. faba* cv. minor (sample 7), the presence of bean yellow mosaic virus (BYMV, Potyvirus genus, Potyviridae family) was detected. In sample 1, which came from *R. pseudoacacia*, the peanut stunt virus (PSV, Cucumovirus genus, Bromiviridae family) was mapped to reference sequences. 

The obtained results of some of the analysed samples were confirmed by standard Sanger sequencing, which also allows to complement HTS contigs to obtain complete viral genomes. Complete genome sequences of identified viruses using HTS were placed in the GenBank, under the accession numbers: MT153866-MT153870, MT130394 and MT176428.

### 2.4. Sequence Analysis of ClYMV and MYFV

**Figure 4:**
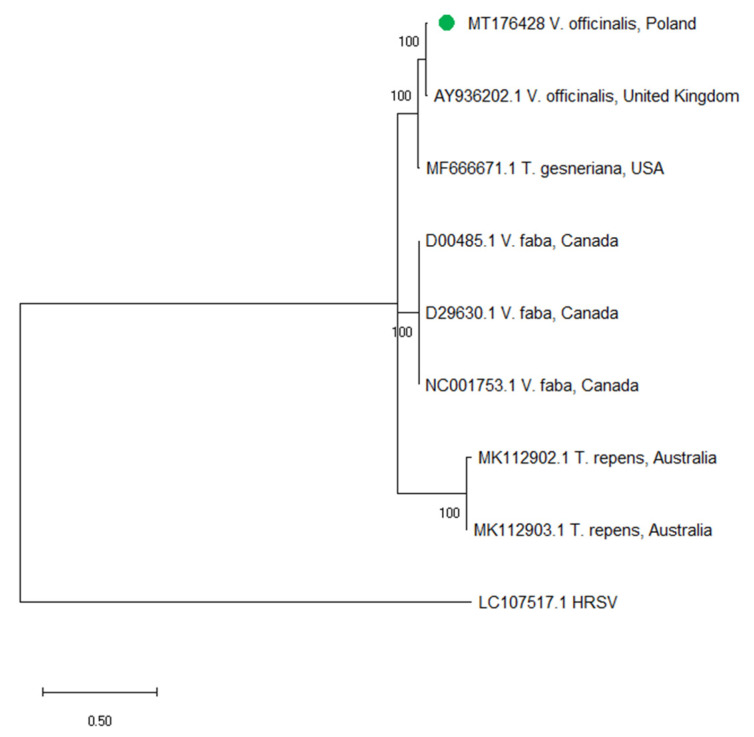
Phylogenetic tree based on the partial coat protein gene (CP) sequences of ClYMV-2018/1 obtained in this study and 7 retrieved from the GenBank. The Polish isolate was marked with green dot (MT176428). Hydrangea ringspot virus (LC107517.1) was used as the outgroup. The tree was constructed by maximum likelihood (ML) method (1000 bootstrap replicates) in MEGA X using Hasegawa–Kishino–Yano model with invariable sites (HKY+I). An accession number, host plant and the country of origin are given for each isolate.

The isolate ClYMV-2018/1 (MT176428) was collected in 2018 in the Wielkopolska region of Poland. The full length genome sequence of ClYMV-2018/1 was compared with the only one available complete RNA sequence of ClYMV isolate originated from Canada (NC001753). The Polish and Canadian isolates shared overall 80.8% nucleotide identity (nt). A comparison of particular open reading frames (ORFs) was also performed. The analysis revealed the following nucleotide (nt) and amino acid (aa) identities: 79.4% and 88.6% for RNA dependent RNA polymerase (RdRp), 82.5% and 90.3% for triple gene block 1 (TGB1), 87.8% and 89.4% for TGB2, 88.7% and 88.1% for TGB3, 83.7% and 92.9% for coat protein gene (CP). In order to obtain the knowledge of the genetic diversity of the ClYMV population, a phylogenetic analysis was performed based on the short fragment (375 nt) of the CP gene of the ClYMV isolate obtained in this study and others described to date. The analysis revealed that the Polish isolate grouped together with the isolate from the United Kingdom (Figure 4). 

The full length RNA1-3 sequences of the newly detected Polish isolate MYFV–2018/1 were compared with the only one available genome sequence (RNAs1-3) of MYFV (AB444583–AB444585). The nucleotide sequence identity between the Polish and Hugarian isolate collected from Melandrium album was 94.8% for RNA 1, 91.6% for RNA 2 and 91.1% for RNA 3, respectively. For better insight of genetic diversity between both isolates, the particular ORFs were also compared. The analysis revealed the following nucleotide (nt) and amino acid (aa) identities: 94.8% and 98.5% for 1a gene, 91.4% and 95.6% for 2a gene, 91.2% nt and 91.8% for 3a gene, 92.4% and 99.4% for CP.

### 2.5. Phylogenetic Analysis of CABYV

**Figure 5:**
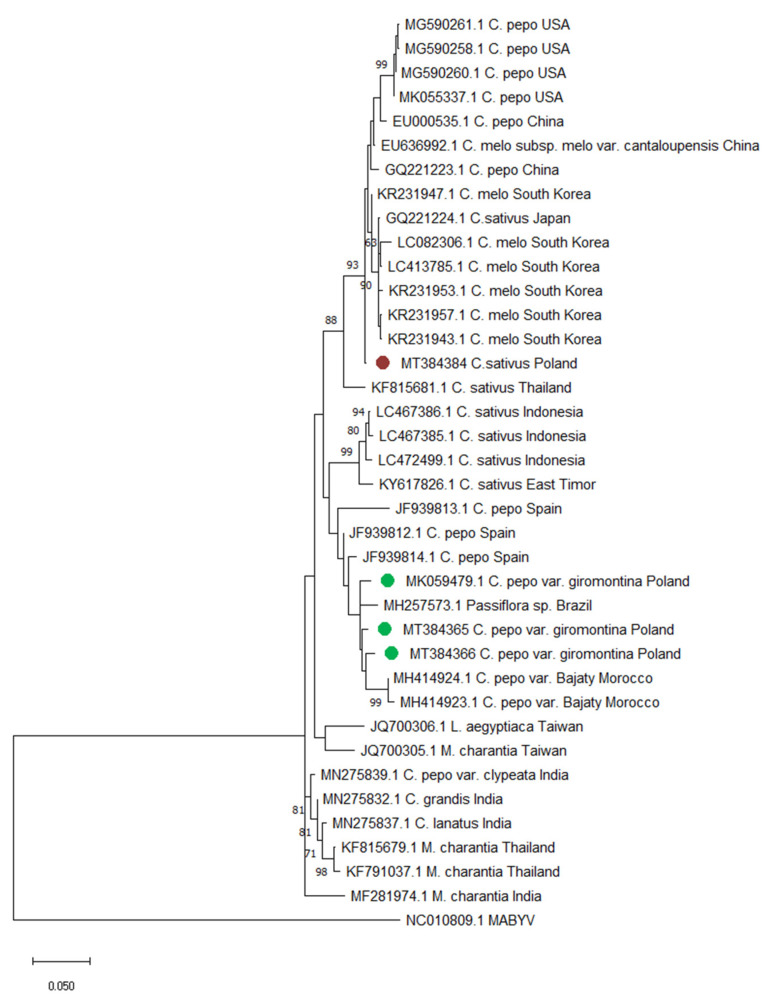
Phylogenetic tree based on the CP sequence of 4 CABYV isolates obtained in this study and 34 retrieved from the GenBank. Isolates collected in Poland in 2018 were marked with green dots (MT384365-66, MK059479); isolates collected in 2019 were marked with red dots (MT384364). Melon aphid-borne yellows virus (NC010809.1) was used as the outgroup. The tree was constructed by maximum likelihood (ML) method (1000 bootstrap replicates) in MEGA X using Kimura-2-parameter model with gamma distribution (G). An accession number, host plant and the country of origin are given for each isolate.

The phylogenetic analysis was performed to obtain an information about the phylogenetic relationships of CABYV isolates from this study with other sequenced isolates of this virus. In the analysis, four Polish CABYV isolates were used; full length genome sequences of three of them were obtained in this study (MT384364, MT384365 and MT384366), whereas one (MK059479) was obtained in the previous experiments [31]. The isolate CABYV-2019/1 (MT384364) was collected in 2019, whereas two isolates: CABYV-2018/1 (MT384365) and CABYV-2018/2 (MT384365) were collected in 2018. The Polish isolates collected in 2018 and 2019 originated from the Wielkopolska region in Poland. The gene encoding coat protein was selected for analysis. The phylogenetic analysis revealed the high diversification of CABYV isolates with the nucleotide identity of CP sequences ranging from 92.2 % to 99.8 %. The presence of recombination events was not detected in the analysed CABYV sequences. The Polish isolates collected in 2018 (MT384365 and MT384366, and MK059479 from the previous study) grouped together with isolates from Spain, Morocco and Brazil, whereas the fourth of the Polish CABYV isolates (MT384364, collected in 2019) clustered together with isolates from China, South Korea, Japan and the USA. Although, the constructed phylogenetic tree showed the presence of two main clusters, no clear division by host plant or country of origin was observed (Figure 5). 

## 3. Discussion 

In the present study, we used different diagnostic methods to detect plant viruses from crops, ornamental plants, weeds, trees and shrubs. An important issue in the disease management is the limited availability of appropriate diagnostic methods. In fact, effective detection protocols are required for maintaining healthy planting material. Standard diagnostic tests, despite potentially high sensitivity and specificity, require specific prior knowledge of the target pathogens, and thus are not suitable for detection of unknown or unexpected pathogens. Moreover, their high specificity might limit the detection of different isolates, variants or strains of particular pathogens, which can lead to false negative results [32,33]. Given the magnitude of plant-infecting pathogens and the frequent occurrence of viruses in mixed infections, it is thus expected that some viruses are missed when using routine targeted diagnostic tests. Here, we used a HTS-based approach, to detect viruses in samples, which were previously tested to be negative using an array of selected targeted RT-PCR tests, but showed positive results using nonspecific TEM and bioassay-based approaches. A total of 13 viruses from different families were identified in 20 tested samples, some of which are rare or have never been found in Poland before. Some of the viruses (CMV, WMV, ZYMV) were detected using HTS, despite a previous negative result in RT-PCR. One of these viruses, CMV, using HTS has been detected in many analysed samples (in 9 of 20 samples). It may indicate the appearance of new genetic variants in nature or a low concentration of viruses in the tested samples.

In this study, we identified two new viruses which originated from weeds: ClYMV and MYFV. The first one, ClYMV infects a broad bean, pea, alfalfa, fat hen, chickweed, and tulips [34,35,36]. Moreover, it causes an important disease of clovers in the United States and Canada [36]. In Europe, it does not pose a significant threat to crops, although it has been identified in the United Kingdom [35] and the Czech Republic [37]. ClYMV belongs to *Potexvirus* genus within Alphaflexiviridae family. Its genome consists of single-stranded plus RNA about 7000 nt in length. The phylogenetic analysis revealed that the Polish isolate is closely related to the isolate from the United Kingdom. The second one—MYFV—belongs to the *Bromovirus* genus of the *Bromoviridae* family. Bromoviruses have three positive-sense RNAs as their genome, designated RNA1, RNA2, and RNA3 [38]. MYFV occurs sporadically and there is only one sequence deposited in the GenBank. The nucleotide sequence identity between the Polish isolate and Hungarian originated from *M. album* ranged between 91.1–94.8% for the genomic RNA1-3. ClYMV and MYFV were detected in weeds that can serve as alternative hosts and potential sources of virus infection. In addition, many wild plants are often infected with more than one viral species at the same time [11]. Surprisingly, when analysing weed plants using HTS, we only detected the presence of single viral infections. Perhaps this is the result of collecting the samples in urban locations, where the presence of potential vectors and the possibility of mechanical viral transmission is more limited.

We have shown that plants from the *Cucurbitaceae* family sampled in Poland are infected with a large spectrum of viruses in mixed infections, including CMV, ZYMV, WMV, CLSV and CABYV. Cucurbits are the major vegetables cultivated worldwide and are affected by more than 70-well-characterised viruses belonging to the main plant virus groups [39]. Most are transmitted by aphids, which in combination with global warming, can promote the faster spread of viruses and the appearance of more severe infections, as well as the horizontal transmission of the viruses to new hosts by their vectors [40]. In 4 of the 11 cucurbit samples, we identified the presence of CABYV. The virus was detected in Poland for the first time in 2018 on zucchini crops in the Wielkopolska region [31]. This was the first report of the occurrence of CABYV in Poland, and the virus has not been previously detected using conventional RT-PCR. CABYV infects cucumber, melon, squash and watermelon, and has also been detected in many weed species, which may be efficient reservoirs [39]. The virus is efficiently transmitted in a persistent, circulative manner by a few aphid species (*Aphis gossypii, M. persicae* and *Macrosiphum euphorbiae*). The abundance of CABYV reservoirs found around cultivated fields and the large populations of *A. gossypii* and *M. persicae* vectors indicated the potential for virus spread, and represents a serious threat to cucurbit production in Poland. It has been shown that CABYV is one of the most common cucurbit viruses in open-field crops, distributed worldwide [41]. Increasing international travel and the trade of plant material enhances the risk of introducing new viruses and their vectors into production systems. It is very likely that, due to the similarity of symptoms induced by CABYV with those induced by other viruses, the presence of the CABYV in Poland remained unnoticed for some years. Our phylogenetic analysis showed geographical and host divergence of CABYV isolates. The Polish isolates of CABYV clustered with isolates from Spain or Brazil, as well as with isolates originating from Asian countries. No clear division by host plant on phylogenetic tree may suggest that CABYV isolates can easily adapt to ever-changing environmental conditions and different hosts.

We have also identified the presence of CLSV in complex with other viruses on zucchini plants. This is the second study reporting the presence of this pathogen in our country [42]. The virus is rarely observed in crops, but it can cause symptoms in the form of chlorotic spots with necrotic centers, slight stunting and the delay of flowering [42,43]. Mixed infections were also identified in the case of tomato crops, where the complex of PepMV and CMV or PepMV and TYRV (tomato sample that gave an unusual pattern of symptoms on infected plants) was observed in tested samples. PepMV is one of the most dangerous viruses that currently infects tomato plants in many European countries, North and South America and China [44,45,46,47,48]. In Poland, it has been widely distributed since 2005. PepMV causes a wide spectrum of symptoms on infected plants, leading to a lower quality and quantity of crops [49,50,51,52,53,54,55,56]. There are various pathotypes of the virus, including necrotic ones, which can lead to plant death. In addition, the presence of the virus in mixed infections with other viruses can lead to higher crop losses. In recent years, the severe damages caused by mixed infections of the viruses belonging to the *Tospovirus* genus: tomato spotted wilt virus and TYRV were observed in tomato crops in Poland [57]. The virus can cause brown necrotic spots on leaves and stems, as well as necrotic spots on fruits. TYRV is relatively rare in Europe. CMV is a pathogen with a very wide host range. On tomatoes, the virus causes the inhibition of growth and stunting of plants, leading to a reduced crop yield. The presence of TYRV and CMV in a mixed infection with PepMV, especially due to their easy mechanical transmission, may pose a serious threat to tomato crops. The presence of mixed virus infections can modulate the symptoms, transmission and pathogenicity [6,7,8,9]. The simultaneous occurrence of different viruses on infected plants can stimulate the emergence of new genetic variants, and thus affect the degree of genetic diversity of their populations [58]. Such occurrence of mixed infections in plants can affect the evolutionary dynamics of the virus or virus population, change the population structure and, therefore, can contribute to stronger and more frequent infections.

In summary, we detected a wide spectrum of pathogens using the HTS-based approach. We have shown that HTS technology is appropriate for the detection of viruses in plants in the absence of prior information on the type of pathogen and its genome structure, or in the case of unclear symptoms of infection. Moreover, we confirmed the potential of HTS for use in the identification of all viruses in mixed infections, including those that could escape detection by RT-PCR. The use of HTS for virus detection will improve the current diagnostic methods, by thoroughly investigating the genomic sequence and the variability of known pathogens, and by designing new diagnostic tools for new viruses.

## 4. Materials and Methods 

### 4.1. Sample Collection

During the growing season (June–September) in 2018 and 2019, a total of 50 plants with different symptoms were collected from different regions in Poland. Part of the samples were provided from the Plant Disease Clinic IPP-NRI. The material consisted of various crops (*S. lycopersicum*, *C. pepo* convar. giromontiina, *C. sativus*, *V. faba* cv. minor), trees and shrubs (*R. pseudoacacia*, *S. nigra*), ornamental plants (*D. belladonna*, *C. multiflorum*, *G. jamesonii*), and weeds (*S. latifolia*, *R. × prostrata*, *V. officinalis*). On the collected plants, various disease symptoms, in the form of leaf blade deformations of varying severity, discoloration, chlorosis, necrosis of leaf blade, and growth reduction, were observed.

### 4.2. Bioassay and Electron Microscopy

All the collected samples were transferred by mechanical inoculation on carborundum dusted test plants: *C. quinoa*, *N. benthamiana*, *N. tabacum* cv. Xanthi, *S. lycopersicum and C. pepo* convar. giromontiina. Leaf fragments (in an amount of 500 mg per plant) were ground in 2 mL of 0.05 M phosphate buffer (pH 7.2) and the obtained sap was applied on tested plants [59]. Plants were maintained under greenhouse conditions at a temperature of 22–23 °C and a photoperiod of 16 h, and the presence of the symptoms was observed for 20 days. 

Subsequently, the presence of the viruses was checked by the standard transmission electron microscope (TEM) procedure. Leaf samples from all the infected plants were crushed in distilled water and applied to Formvar coated copper grids (Polysciences, Warrington, UK). Then, the grids were dyed with ammonium molybdate (MA) or phosphotungstic acid (PTA) and dried [59]. The presence of the viral particles in each sample was checked by an HT7700 TEM microscope (Hitachi, Japan), at an accelerating voltage of 80 kV.

### 4.3. RT-PCR Detection

Total RNAs were isolated from all collected plants and from test plants 10 days post inoculation (dpi), using RNeasy Plant Mini Kit (Qiagen, Hilden, Germany), according to the manufacturer’s protocol, and dissolved in 50 μL of sterile water. Then, RNAs were measured fluorometrically using a Qubit 3 fluorometer (Thermo Fisher Scientific, Waltham, MA, USA). The presence of the selected (most common) viruses in each sample was tested using a Transcriptor One-Step RT-PCR Kit (Roche, Mannheim, Germany), according to the manufacturer’s procedure. Plants from the *Cucurbitaceae* family were tested for the presence of CMV, CABYV, cucumber green mottle mosaic virus (CGMMV, *Tobamovirus* genus, *Virgoviridae* family), papaya ringspot virus (PRSV, *Potyvirus* genus, *Potyviridae* family), TBRV, WMV and ZYMV. Tomato plants were tested for PepMV, PVY, and CMV, whereas *V. faba* cv. minor was checked for broad bean true mosaic virus (BBTMV, *Comovirus* genus, *Secoviridae* family) and BYMV. Ornamental plants and trees were tested for the presence of viruses from the *Nepovirus* genus using the primers for nepo A, nepo B and nepo C groups. Moreover, gerbera plants were tested on TSWV and chrysanthemum plants on *Chrysanthemum virus B* (CVB, *Carlavirus* genus, *Betaflexiviridae* family) and TSWV. All the primers used in the experiment are summarised in Table 2 [60,61,62,63,64,65,66,67,68,69,70,71,72]. The resulting PCR products were separated on 1% agarose gel to verify the appropriate size. Then, the products were purified using NucleoSpin® Gel and PCR Clean-up (Macherey-Nagel, Düren, Germany), according to the manufacturer’s protocol, and sequenced using the standard Sanger procedure.

### 4.4. Preparing the Samples for High-throughput Sequencing

The next generation sequencing procedure was performed for 20 samples (Table 1). For HTS, mainly RNAs isolated from collected plants were used. In some cases, the quality of collected material, and therefore, the concentration of RNAs extracted directly from collected material, was not sufficient for HTS, so in these cases, the RNAs isolated from symptomatic test plants were used. The quality of RNA was estimated using capillary electrophoresis Qsep-100 DNA Analyzer (BiOptic Inc., Taipei, Taiwan). The next generation sequencing of the first 11 samples was performed on the Illumina NextSeq500 platform, with 36 nt paired-end chemistry by Genomed S.A company (Warsaw, Poland). Ribosomal RNA was depleted from the purified RNA using the RiboMinus Plant Kit for RNA-Seq (Thermo Fisher Scientific, Waltham, MA, USA), and sequencing libraries were prepared using NEBNext Ultra Directional RNA Library Prep Kit for Illumina (New England Biolabs, Ipswich, MA, USA). Subsequently, the library of the other 9 samples was prepared using TruSeq stranded total RNA with the RiboZero Plant kit (Illumina, San Diego, CA, USA), while high-throughput sequencing was performed on the Illumina NovaSeq 6000 platform, with 100 nt paired-end chemistry by CeGaT company (Tübingen, Germany). The bioinformatics analysis of the obtained raw data was performed using the CLC Genomics Workbench (Qiagen, Hilden, Germany). Reads obtained in 2018 were trimmed and filtrated (reads with Q ≤ 25 and shorter than 15 bp were discarded) by an external company using CLC Genomics Workbench 7.0.4, while adapters from reads from 2019 sequencing were trimmed with Skewer (version 0.2.2) [73]. The quality was analysed with FastQC (version 0.11.5-cegat) [74]. Trimmed sequencing reads of separate samples were first mapped to NCBI viral RefSeq database (January 2019 for the first batch of samples and February 2020 for the second batch of samples). The minimum percentage of the required total alignment length matching the reference sequence at the selected similarity fraction was set at 50%, while the minimum percentage identity between the aligned region of the read and the reference sequence was set to 80%. For positive hits (the highest average depth of coverage for corresponding viral species), closest reference sequences were selected from the NCBI GenBank database (January 2019 and February 2020, respectively), according to the blastN similarity searches of the consensus sequences generated by the first mappings. Reads were then mapped as described above, to the corresponding most similar viral genome sequences from NCBI GenBank (shown in Table 1), and results were reported in the form of the table (Table 1). Consensus viral genome sequences were extracted from these mappings and reads of corresponding samples were again mapped to these consensus sequences. The mappings were visually inspected for possible mismatches, to ensure the quality of the reconstructed consensus viral genomes. Finally, the consensus viral genomes were deposited in NCBI GenBank and used for further analyses.

### 4.5. Confirmation of Obtained Results by RT-PCR and Sanger Sequencing

The presence of selected viruses detected by HTS was checked by standard RT-PCR procedure and Sanger sequencing. The presence of the viruses in each sample was checked using a Transcriptor One-Step RT-PCR Kit (Roche, Mannheim, Germany), according to the manufacturer’s procedure, with a set of specific primers designed based on the consensus sequences obtained by HTS (Table 3). PCR products were separated on a 1% agarose gel to verify the appropriate size and purified by NucleoSpin® Gel and PCR Clean-up (Macherey-Nagel, Düren, Germany), according to the manufacturer’s procedure. The PCR products were sequenced by Genomed S.A. Company (Warsaw, Poland). 

### 4.6. Sequence Analysis of ClYMV and MYFV

The full-length genome sequence of ClYMV-2018/1 was compared with the one genome sequence of ClYMV available in GenBank (NC001753). The comparison of particular ORFs was also performed using the Sequence Identity Matrix in BioEdit software [75]. The phylogenetic analysis of ClYMV isolates was conducted using the fragment of CP gene sequence of the isolate obtained in this study (ClYMV-2018/1) and 7 others deposited in GenBank. Moreover, HRSV (LC107517.1) was used as the outgroup. The information about particular isolates (accession number, host plant and country of origin) was placed on the tree. 

The nucleotide sequences were aligned by codon using MUSCLE algorithm [76], as implemented in MEGA X [77]. The phylogenetic tree was constructed by the maximum likelihood (ML) method in MEGA X, using the Hasegawa–Kishino–Yano model with invariable sites (HKY+I) as the best-fitting one based on the lower BIC [78]. Bootstrap values were calculated using 1000 random pseudoreplicates.

The genomic RNAs of MYFV-2018/1 isolate obtained in this study were compared with one full-length genome sequence of MYFV deposited in GenBank. The Hungarian isolate of MYFV originated from M. album (AB444583-AB444585). Nucleotide sequence identity of RNA1-RNA3 was determined using the Sequence Identity Matrix in BioEdit software [75].

### 4.7. Recombination and Phylogenetic Analyses of CABYV

The phylogenetic analyses were conducted using the gene encoding coat protein (CP) sequences of 37 CABYV isolates, both obtained by us in this experiment, as well as those deposited in GenBank. Moreover, MABYV (MC010809.1) was used as the outgroup. The information about particular isolates (accession number, host plant and country of origin) were placed on the tree. 

The CP nucleotide sequences were aligned by codon using MUSCLE algorithm [76], as implemented in MEGA X [77]. Subsequently, the presence of recombinants in the analysed CABYV population were investigated by the RDP4 package. The recombination-detecting algorithms GENCONV, BootScan, MaxChi, Chimaera, SiScan, 3Seq and RDP were used, and recombination events were considered as significant if four or more methods had a P<0.05. The phylogenetic tree was constructed by maximum likelihood (ML) method in MEGA X, using Kimura-2-parameter model with gamma distribution (G) as the best-fitting one based on the lower BIC [78]. Bootstrap values were calculated using 1000 random pseudoreplicates.

## Figures and Tables

**Table 1:** All identified viruses using high throughput sequencing (HTS)-based approach with a corresponding sample number, original host plant species, test plant species, total number of reads, the number of reads mapped for the individual virus reference sequences for each analysed sample, average depth of coverage, and percent of reference genome covered by reads.

No	Host Plant	Plant Used for RNA Isolation	Number of Total Raw Reads	Percent of Reference Genome Covered by Reads	Number of Reads Mapped to Corresponding Reference Sequence from Viral RefSeq	Average Depth of Coverage for Corresponding Viral Species	Identified Viruses
**1**	*R. pseudoacacia*	*N. benthamiana*	11,027,620	100% 83.05% 64.64% 100%	41,097 67,428 16,277 71,540	3629.22 730.04 201.40 1139.87	satRNA peanut stunt virus (NC_003855) * RNA1 peanut stunt virus (NC_002038) RNA2 peanut stunt virus (NC_002039) RNA3 peanut stunt virus (NC_002040)
**2**	*V. officinalis*	*C. quinoa*	98,173,277	90.27%	401,558	1945.10	clover yellow mosaic virus (NC_001753)
**3**	*D. belladonna*	*N. benthamiana*	46,911,057	91.73% 97.48%	63,133 92,157	295.03 837.80	RNA1 arabis mosaic virus (NC_006057) RNA2 arabis mosaic virus (NC_006056)
**4**	*C. pepo*	*C. pepo*	18,034,634	99.46% 93.72% 97.49%	41,742 21,359 3140	154.49 75.70 19.60	zucchini yellow mosaic virus (NC_003224) watermelon mosaic virus (NC_006262) cucurbit aphid-borne yellows virus (NC_003688)
**5**	*C. pepo*	*C. pepo*	600,806	99.94% 99.73% 99.86% 97.61% 67.7% 72.57%	19,301 19,814 53,922 1238 1361 205	341.50 340.47 1293.96 7.47 2.19 1.91	RNA1 cucumber mosaic virus (NC_002034) RNA2 cucumber mosaic virus (NC_002035) RNA3 cucumber mosaic virus (NC_001440) zucchini yellow mosaic virus (NC_003224) watermelon mosaic virus (NC_006262) cucurbit aphid-borne yellows virus (NC_003688)
**6**	*C. pepo*	*C. pepo*	56,369,111	91.8% 67.29% 84/09% 97.15%	59,518 206 198 488	206.72 2.1 2.26 7.84	watermelon mosaic virus (NC_006262) RNA1 cucumber mosaic virus (NC_002034) RNA2 cucumber mosaic virus (NC_002035) RNA3 cucumber mosaic virus (NC_001440)
**7**	*V. faba*	*N. benthamiana*	19,979,239	85.62%	186,607	700.15	bean yellow mosaic virus (NC_003492)
**8**	*S. latifolia*	*N. benthamiana*	302,784	99.63% 98.25% 99.21%	71,934 25,849 7627	1662.62 708.24 250.10	RNA1 melandrium yellow fleck virus (NC_013266) RNA2 melandrium yellow fleck virus (NC_013267) RNA3 melandrium yellow fleck virus (NC_013268)
**9**	*C. pepo*	*C. pepo*	35,797,184	96.79% 94.45% 95.43%	41,613 12,029 3567	152.35 42.04 21.99	zucchini yellow mosaic virus (NC_003224) watermelon mosaic virus (NC_006262) cucurbit aphid-borne yellows virus (NC_003688)
**10**	*C. pepo*	*C. pepo*	26,025,392	97.09%	74,858	261.66	watermelon mosaic virus (NC_006262)
**11**	*C. sativus*	*C. sativus*	773,010	99.91% 99.44% 99.77%	10,882 20,570 40,511	157.57 321.49 880.88	RNA1 cucumber mosaic virus (NC_002034) RNA2 cucumber mosaic virus (NC_002035) RNA3 cucumber mosaic virus (NC_001440)
**12**	*C. pepo*	*C. pepo*	32,033,000	99.32%	18,647,504	185,152.26	watermelon mosaic virus (NC 006262)
**13**	*C. pepo*	*C. pepo*	31,632,000	99.16% 99.58% 99.37% 99.09%	4,166,267 33,803 42,961 58,722	41,361.90 1006.61 1387.22 2573.19	watermelon mosaic virus (NC_006262) RNA1 cucumber mosaic virus (NC_002034) RNA2 cucumber mosaic virus (NC_002035) RNA3 cucumber mosaic virus (NC_001440)
**14**	*C. sativus*	*C. sativus*	30,564,000	99.85% 100% 99.9% 98.07% 99.21%	4,179,988 4,387,800 11,630,859 113,703 4396	123,854.64 144,669.22 527,062.54 1987.71 14.38	RNA1 cucumber mosaic virus (NC_002034) RNA2 cucumber mosaic virus (NC_002035) RNA3 cucumber mosaic virus (NC_001440) cucurbit aphid-borne yellows virus (NC_003688) cucumber leaf spot virus (NC_007216)
**15**	*C. pepo*	*C. pepo*	31,237,000	100% 100% 99.61% 98.55% 100%	7,434,123 4,038,168 24,208 31,393 39,424	77,638.71 40,148.47 722.10 1017.05 1740.52	zucchini yellow mosaic virus (NC_003224) watermelon mosaic virus (NC_006262) RNA1 cucumber mosaic virus (NC_002034) RNA2 cucumber mosaic virus (NC_002035) RNA3 cucumber mosaic virus (NC_001440)
**16**	*R. × prostrata*	*N. benthamiana*	29,986,000	97.49% 89.90% 98.45% 96.93%	5,884,878 311 5015 602	58,322.65 9.25 16.63 26.44	turnip mosaic virus (NC_002509) RNA1 cucumber mosaic virus (NC_002034) RNA2 cucumber mosaic virus (NC_002035) RNA3 cucumber mosaic virus (NC_001440)
**17**	*S. lycopersicum*	*S. lycopersicum*	38,560,000	98.48% 99.95% 97.88% 94.87%	29,548,236 1,297,591 2,472,631 2,594,718	433,514.97 14,364.57 50,882.36 85,169.46	pepino mosaic virus (NC_004067) Segment L tomato yellow ring virus (JN 560178) Segment M tomato yellow ring virus (JN 560177) Segment S tomato yellow ring virus (DQ 462163)
**18**	*C. pepo*	*C. pepo*	36,021,000	100% 100% 100%	8,440,062 12,232,893 35,606,110	250,241.34 403,564.26 1,612,531.81	RNA1 cucumber mosaic virus (NC_002034) RNA2 cucumber mosaic virus (NC_002035) RNA3 cucumber mosaic virus (NC_001440)
**19**	*S. lycopersicum*	*S. lycopersicum*	36,694,000	99.94% 99.73% 99.68% 99.98%	1,565,570 1,386,012 6,284,859 24,007,721	499,564.13 820,381.91 1,764,993.94 116,343.05	RNA1 cucumber mosaic virus (NC_002034) RNA2 cucumber mosaic virus (NC_002035) RNA3 cucumber mosaic virus (NC_001440) pepino mosaic virus (NC 004067)
**20**	*S. lycopersicum*	*S. lycopersicum*	39,382,000	100% 100% 99.68% 99.81%	13,891,631 21,749,691 34,738,425 6438	412,119.76 704,894.27 1,539,186.77 49.33	RNA1 cucumber mosaic virus (NC_002034) RNA2 cucumber mosaic virus (NC_002035) RNA3 cucumber mosaic virus (NC_001440) pepino mosaic virus (NC_004067)

* Accession number of sequences from RefSeq viral genomes database.

**Table 2:** Primers used in RT-PCR reactions.

Virus	Primer	Sequence 5′-3′	Reference
cucumber mosaic virus	CMV CPf CMV CPr	GCTTCTCCGCGAG GCCGTAAGCTGGATGGAC	[60]
cucurbit aphid-borne yellows virus	CABYVCPF CABYVCPRev	ATGAATACGGCCGCGGCTAGAAATC CTATTTCGGGTTCTGGACCTGGCA	[61]
cucumber green mottle mosaic virus	CGMMV-F5370 CGMMV-R6390	CTAATTATTCTGTCGTGGCTGCGGATGC CTTGCAGAATTACTGCCCATA	[62]
papaya ringspot virus	04-02 04-04	TACTAGTGTACCATGAATC CTCTCATTCTAAGAGGCTC	[63]
tomato black ring virus	TBRV CPF TBRV CPR	GCCTGTCTCTCTCGCAATG AAGGAGCCAAACTGAAATG	[64]
watermelon mosaic virus	WMV F WMVR	GAA TCA GTG TCT CTG CAA TCA GG ATT CAC GTC CCT TGC AGT GTG	[65]
zucchini yellow mosaic virus	ZY-1, ZY-2	CACAATTTTCCCATGAGAACCAGC GCTCCATACATAGCTGAGACAGC	[66]
pepino mosaic virus	TGB3F TGB3R	GGTGGACAATATCAAGACCGG CTGTATTGGGTTTGAGAAGTC	[67]
potato virus Y	PVYc3 PVYf PVY3+ PVY3− CP2+ CP1−	CAACGCAAAAACACTCA(CT)AAA(AC)GC TAAGTG(AG)ACAGACCCTCT(CT)TTCTC TGTAACGAAAGGGACTAGTGCAAAG CCGCTATGAGTAAGTCCTGCACA CCAGTCAAACCCGAACAAAGG GGCATAGCGTGCTAAACCCA	[68]
broad bean true mosaic virus	BBTMV-IGGf BBTMV-VQTr	CnAThGGnGGnGGnGCnGG CACyTGnGTnGACCAnGC	[69]
bean yellow mosaic virus	BYMV-CP-5 BYMV-CP-3	GAACTGTTGGAACGTTTTCAATTCC TCTGTTCCAACATTGCCATCAAG	
*Nepovirus* genus	Nepo-AF Nepo-AR Nepo-BF Nepo-BR Nepo-CF Nepo-CR	GGHDTBCAKTMYSARRARTGG TGDCCASWVARYTCYCCATA ATGTGYGCHACYACWGGHATGCA TTCTCTDHAAGAAATGCCTAAGA TTRKDYTGGYKAAMYYCCA TMATCSWASCRHGTGSKKGCCA	[70]
tomato spotted wilt virus	TS1-F TS1-R	GCCTATGGATTACCTCTTG GTTTCACTGTAATGTTCCA	[71]
chrysanthemum virus B	CVB-F CVB-R	AGTCACAATGCCTCCCAAAC CATACCTTTCTTAGAGTGCTATGCT	[72]

**Table 3:** Primers used for RT-PCR reaction and Sanger sequencing to confirm the HTS results.

Virus	Primer	Sequence 5′-3′	Amplified Region of the Genome	Amplicon Size [bp]	Reference
TYRV	TYRVLF1473 TYRVLR2068	GGAGAAATGAATTTTAA CTTTGTATCATTGAAT	RdRp	595	This study
CIMYV	ClMYVF1574 ClMYVR2620	CAAGTCCTGAACAGAGT AGTTTCCAGGGTAGTTC	RdRp	1046	This study
TuMV	TuMVF1194 TuMVR2108	TGAGCCATAAGATTGTGCAT AGTGGATCACCTGATTC	MP	914	This study
MYFV	MYFMV2F2577 MYFMV2R2840	CTAAGTAAGTTGCTAATGC GGTCTCCTTTATGACCACTAATC	2a/3’UTR	263	This study

## Supplementary Materials

Table S1 is available online at https://www.mdpi.com/2223-7747/9/7/820/s1, Table S1: Results of bioassay and RT-PCR reactions of all collected samples.
