# Supplementary material for: High-Throughput Sequencing Facilitates Discovery of New Plant Viruses in Poland
Source: Plants (Basel). 2020 Jun 29;9(7):820. doi: 10.3390/plants9070820 (PMC7411967; doi:10.3390/plants9070820)
Supplement: Supplementary file 1 [file plants-09-00820-s001.pdf]

Sample	Host Plant	GPS**	Symptoms on infected plants***						Viruses identified by RT-PCR
			Original plants	*N. benthamiana*	*S. lycopersicum*	*C. quinoa*	*N. tabacum*	*C. pepo*	
**1**	***R. pseudoacacia***	52°53′N 17°61′E	cm, ls	-	Not tested	-	-	Not tested	-
**2**	***R. pseudoacacia***	52°53′N 17°61′E	cm, ls,	-	Not tested	lns, nr	nr, ld	Not tested	TBRV, NepoB
**3**	***R. pseudoacacia***	52°53′N 17°61′E	cm, ls, ld	-	Not tested	-	-	Not tested	-
**4**	***R. pseudoacacia***	52°53′N 17°61′E	cm, sm	-	Not tested	lns, nr	nr, ld	Not tested	TBRV, NepoB
**5**	***R. pseudoacacia***	52°53′N 17°61′E	cm, ls, ld	-	Not tested	-	-	Not tested	-
**6**	***R. pseudoacacia****	52°53′N 17°61′E	lc, ls,, ld	-	Not tested	lcs	-	Not tested	-
**7**	***R. pseudoacacia***	52°53′N 17°61′E	cm, ls,	-	Not tested	lns, nr	nr, ld	Not tested	TBRV, NepoB
**8**	***R. pseudoacacia***	52°53′N 17°61′E	cm, ls,	-	Not tested	lns, nr	nr, ld	Not tested	TBRV, NepoB
**9**	***V. officinalis****	-	gr, s, ld	-	-	cs	-	Not tested	Not tested
**10**	***S. latifolia****	52°75′ N 16°99′E	gr, s, ld	lc, ld	-	-	-	Not tested	Not tested
**11**	***R. × prostrata****	52°23'09.9"N 16°51'24.1"E	ls, gr, ld	ld	-	-	-	Not tested	Not tested
**12**	***D. belladonna****	-	ld	cm, ld	-	lc	-	Not tested	-
**13**	***V. faba****	-	cm, nl	-	Not tested	cs	-	Not tested	-
**14**	***V. faba***	-	cm	-	Not tested	-	-	Not tested	-
**15**	***S. nigra***	52°75′ N 16°99′E	cm	-	Not tested	-	-	Not tested	-
**16**	***S. nigra***	52°75′ N 16°99′E	cm	-	Not tested	lns, nr	nr, ld	Not tested	TBRV, NepoB
**17**	***S. nigra***	52°75′ N 16°99′E	cm	-	Not tested	-	nr, ld	Not tested	TBRV, NepoB
**18**	***S. lycopersicum***	-	gr, ld,	ld	ld,	-	-	Not tested	PepMV
**19**	***S. lycopersicum***	-	gr, ld, dl	ld	ld,	-	vc	Not tested	PepMV, PVY
**20**	***S. lycopersicum***	-	gr, ld, nl	ld	ld	lcs	cs,	Not tested	PepMV, CMV
**21**	***S. lycopersicum****	-	gr, ld, bns	ld	ld, bns	lcs	cs	Not tested	PepMV
**22**	***S. lycopersicum***	-	gr, ld	ld	ld	-	-	Not tested	PepMV
**23**	***S. lycopersicum****	-	gr, ld, nl, lt	ld	ld, nl	lcs	cs	Not tested	-
**24**	***S. lycopersicum***	-	gr, ld,	ld	ld	-	-	Not tested	PepMV
**25**	***S. lycopersicum****	-	gr, ld, nl, lt	ld	ld, nl	lcs	cs	Not tested	-

Sample	Host Plant	GPS**	Symptoms on infected plants***						Viruses identified by RT-PCR
			Original plants	*N. benthamiana*	*S. lycopersicum*	*C. quinoa*	*N. tabacum*	*C. pepo*	
**26**	***S. lycopersicum***	-	gr, ld	ld	ld	-	-	Not tested	PepMV
**27**	***S. lycopersicum***	-	gr, ld, dl	ld	ld	-	vc	Not tested	PVY
**28**	***C. multiflorum***	-	lns, gr	ld	-	-	-	Not tested	CVB
**29**	***C. multiflorum***	-	lns, gr	ld, cs	lns	lns	lns, nr	Not tested	TSWV
**30**	***C. multiflorum***	-	lns, gr	ld	-	-	-	Not tested	CVB
**31**	***G. jamesonii***	-	nss	ld, cs	lns	lns	nr	Not tested	TSWV
**32**	***G. jamesonii***	-	nss	ld, cs	lns	lns	nr	Not tested	TSWV
**33**	***G. jamesonii***	-	nss	ld, cs	lns	lns	nr	Not tested	TSWV
**34**	***C. sativus***	-	ld, lt, cm, sm	Not tested	Not tested	lcs	cs	cm, sm	CMV
**35**	***C. sativus****	-	ld, lt, cm, sm	Not tested	Not tested	lcs,nl	cs	cm, sm	-
**36**	***C. sativus***	-	ld, lt, cm, sm	Not tested	Not tested	-	-	cm, sm	WMV
**37**	***C. sativus****	-	ld, lt, cm, sm, vc, m	Not tested	Not tested	lcs,nl	ld, dl	cm, sm	-
**38**	***C. sativus***	-	ld, lt, cm, sm	Not tested	Not tested	lcs	cs	cm, sm	CMV
**39**	***C. pepo***	-	ld, lt, cm, sm	Not tested	Not tested	lcs	cs	cm, sm	CMV, WMV
**40**	***C. pepo****	-	ld, lt, cm, sm	Not tested	Not tested	lcs	cs	cm, sm	-
**41**	***C. pepo****	-	ld, lt, cm, sm, vc, m	Not tested	Not tested	lcs	cs	cm, sm	WMV
**42**	***C. pepo****	-	ld, lt, cm, sm	Not tested	Not tested	lcs	cs	cm, sm	-
**43**	***C. pepo****	-	ld, lt, cm, sm, vc, m	Not tested	Not tested	-	-	cm, sm	-
**44**	***C. pepo****	-	ld, lt, cm, sm, m	Not tested	Not tested	-	-	cm, sm	WMV
**45**	***C. pepo****	-	ld, lt, cm, sm, m	Not tested	Not tested	-	-	cm, sm	WMV
**46**	***C. pepo****	-	ld, lt, cm, sm	Not tested	Not tested	lcs	cs	cm, sm	-
**47**	***C. pepo****	-	ld, lt, cm, sm, vc, m	Not tested	Not tested	-	-	cm, sm	-
**48**	***C. pepo****	-	ld, lt, cm, sm	Not tested	Not tested	lcs	cs	cm, sm	-
**49**	***C. pepo***	-	ld, lt, cm, sm	Not tested	Not tested	-	-	cm, sm	WMV, ZYMV
**50**	***C. pepo***	-	ld, lt, cm, sm	Not tested	Not tested	-	-	cm, sm	CMV, WMV, ZYMV

Table S1. Results of bioassay and RT-PCR reactions of all collected samples. Samples that did not show disease symptoms after being transferred to the test plants and were negative in RT-PCR were marked in gray. Plants for which HTS was performed were marked with an asterisk.

**GPS location has been added only for the samples that were collected by employees of the Department of Virology and Bacteriology IPP-NRI. The GPS locations of samples from the Plant Disease Clinic IPP-NRI are confidential.

***Symptoms on original and test plants: lc - leaf chlorosis, cm - chlorotic mosaic, ls - leaf stunting, lns – local necrotic spot, nr - necrotic ringspot, gr - growth reduction, nl - necrotic lesion, cs - chlorotic spots, ld - leaf deformation, nrf - necrotic ringspot of fruits, lt - leaf thickening, sm- severe mosaic, m-mottling, nss - necrotic spot on steam, lcs – local chlorotic spots, vc - vein clearing, bns – brown necrotic spots on on leaves and steem, m- mottling, s – stunting, dl – discoloration of leaves, "-" - no symptoms.
